# Applying Integrated Exposure-Response Functions to PM_2.5_ Pollution in India

**DOI:** 10.3390/ijerph16010060

**Published:** 2018-12-26

**Authors:** Vijay S. Limaye, Wolfgang Schöpp, Markus Amann

**Affiliations:** 1 Nelson Institute for Environmental Studies, Center for Sustainability and the Global Environment (SAGE), University of Wisconsin-Madison, Madison, WI 53726, USA; 2 Department of Population Health Sciences, University of Wisconsin-Madison, Madison, WI 53726, USA; 3 International Institute for Applied Systems Analysis, 2361 Laxenburg, Austria; schoepp@iiasa.ac.at (W.S.); amann@iiasa.ac.at (M.A.)

**Keywords:** air pollution, exposure-response, modeling, health impact assessment, India

## Abstract

Fine particulate matter (PM_2.5_, diameter ≤2.5 μm) is implicated as the most health-damaging air pollutant. Large cohort studies of chronic exposure to PM_2.5_ and mortality risk are largely confined to areas with low to moderate ambient PM_2.5_ concentrations and posit log-linear exposure-response functions. However, levels of PM_2.5_ in developing countries such as India are typically much higher, causing unknown health effects. Integrated exposure-response functions for high PM_2.5_ exposures encompassing risk estimates from ambient air, secondhand smoke, and active smoking exposures have been posited. We apply these functions to estimate the future cause-specific mortality risks associated with population-weighted ambient PM_2.5_ exposures in India in 2030 using Greenhouse Gas-Air Pollution Interactions and Synergies (GAINS) model projections. The loss in statistical life expectancy (SLE) is calculated based on risk estimates and baseline mortality rates. Losses in SLE are aggregated and weighted using national age-adjusted, cause-specific mortality rates. 2030 PM_2.5_ pollution in India reaches an annual mean of 74 μg/m^3^, nearly eight times the corresponding World Health Organization air quality guideline. The national average loss in SLE is 32.5 months (95% Confidence Interval (CI): 29.7–35.2, regional range: 8.5–42.0), compared to an average of 53.7 months (95% CI: 46.3–61.1) using methods currently applied in GAINS. Results indicate wide regional variation in health impacts, and these methods may still underestimate the total health burden caused by PM_2.5_ exposures due to model assumptions on minimum age thresholds of pollution effects and a limited subset of health endpoints analyzed. Application of the revised exposure-response functions suggests that the most polluted areas in India will reap major health benefits only with substantial improvements in air quality.

## 1. Introduction

A wealth of epidemiological research over the past two decades indicates that both acute and chronic exposure to ambient air pollution is associated with adverse health effects [1]. Fine particulate matter (PM_2.5_, particles of aerodynamic diameter ≤2.5 microns) is consistently implicated as the most damaging pollutant to human health. The World Health Organization (WHO) has estimated that ambient air pollution contributes to 3.2 million premature deaths and 76,163 disability adjusted life years annually, largely due to the impacts of cardiovascular disease [2]. Of this staggering total, two-thirds of the burden falls in Asia, where PM_2.5_ concentrations are some of the world’s highest [3]. 

Fossil fuel combustion contributes the majority of hazardous PM_2.5_, at the same time generating earth-warming greenhouse gases. The Greenhouse Gas-Air Pollution Interactions and Synergies (GAINS) model identifies emission control strategies that maximize co-benefits from the control of local air quality, specifically in mitigation of greenhouse gas emissions and reduced human mortality from exposures to air pollution. GAINS utilizes a detailed spatial and sector-specific emissions inventory to calculate annual average ambient concentrations of PM_2.5_ with the use of source-receptor relationships from the TM5 model [4], which calculates PM_2.5_ concentrations at a 1° spatial resolution. The TM5 approach is augmented by a special routine to identify sub-grid differences in PM_2.5_ concentrations as a function of local emission densities and the spatial extensions of urban areas within each grid cell. Through this method, GAINS applies population data from the CIESIN 2.5° database to calculate an urban increment of PM_2.5_ that can be applied to major population centers [4]. 

The Indian subcomponent of GAINS encompasses 553 1° grid cells, and also considers transboundary air pollution spanning neighboring countries. PM_2.5_ pollution in India is primarily sourced to fuel combustion in non-industrial plants and the manufacturing industry [5]. Another important source is solid fuel combustion for cooking and heating in homes; indoor air pollution is a significant source of all air-related health impacts in the country [6,7,8]. Increasing industrialization, urbanization, population growth, and demand for transportation also exacerbate PM_2.5_ concentrations. While the link between indoor PM_2.5_ exposures and health effects is well established [7], there remains a dearth of evidence on the specific sources and chemical properties of PM_2.5_ that are most hazardous [9].

### 1.1. Current GAINS Health Impact Assessment Methodology

Prospective cohort studies largely confined to Western countries provide the epidemiological basis for health impact assessments of PM_2.5_ [10,11]. The GAINS model includes a health impact module that quantifies the effects of chronic exposure to air pollutants (ozone and PM_2.5_) on reduced life expectancy for adults aged 30 and older [12]. Due to the lack of region-specific epidemiologic evidence identifying associations with mid- and high-level PM_2.5_ exposures and health impacts, GAINS applies the PM_2.5_ relative risk function for all-cause mortality derived by Pope et al., (2002) [13], a robust estimate that is in agreement with other long-term studies. However, this methodology does not constrain the accumulation of health impacts related to PM_2.5_ exposures at any level, contrary to the evidence from proxy exposures of a plateau in relative risk the due to a combination of factors (see Section 1.3) [14,15,16,17,18,19,20]. Therefore, the health impact estimates from GAINS and other models like it may not accurately reflect the human health toll of air pollution in highly polluted settings. The limitations of current extrapolation techniques are a focus of attention from pollution regulators and researchers in India, who have called for more investigation into the particular exposures and vulnerabilities of populations in the region [21,22].

### 1.2. All-Cause vs. Cause-Specific Mortality

A review of PM_2.5_-related health studies provides estimates of log-linear concentration-response functions for three categories of cause-specific mortality: cardiovascular disease (ischemic heart disease and cerebrovascular disease/stroke), respiratory disease (acute lower respiratory infection and chronic obstructive pulmonary disease), and lung cancer [3,23]. The relative risks associated with PM_2.5_ exposure as specified in the American Cancer Society’s Cancer Prevention Study-II (CPS-II) are indicated below in Table 1. Compared to the all-cause risk estimate, the relative risk values are higher for cardiovascular disease and lung cancer, due to the fact that the baseline annual mortality incidence rates for these specific outcomes are by definition lower than annual all-cause mortality incidence (see Table 2 for estimates of cause-specific mortality rates in India).

### 1.3. Risk at High Levels of Pollution

Most prospective cohort studies exploring health risks from chronic exposure to PM_2.5_ have been conducted in developed countries, where ambient PM_2.5_ concentrations commonly range between 5–30 µg/m^3^. However, PM_2.5_ levels in developing countries commonly exceed this range [25,26], and current health impact estimation techniques in GAINS do not adequately adjust for this important difference. Estimates of urban air pollution derived from direct monitoring [27,28] and satellite measurements of aerosol optical depth [29,30] indicate that PM_2.5_ levels in Indian cities routinely exceed levels deemed unsafe for human health, according to the country’s national ambient air quality standards and intermediate guideline levels recommended by the WHO [9,27,31,32,33]. To date, no large epidemiological cohort studies have estimated the effect of chronic exposure to PM_2.5_ on risk of death at these high concentrations, though state-specific estimates of health impacts using statistical modeling have recently been published [8].

The most recent Global Burden of Disease (GBD) analysis continued the cause-specific analysis to hypothesize about the relative risk functions at higher doses of PM_2.5_ in ambient air. The assessment posited integrated exposure-response functions for specific causes of death [2]. Under this approach, the source and precise chemical composition of fine particles is of secondary concern to the absolute quantity of inhaled pollution; as a result, health impacts due to exposure from ambient air, secondhand smoke, and active cigarette smoking can be aggregated and analyzed along a unified exposure-response curve [20,34,35]. The integrated exposure-response model is posited because the risk of death is not seen to rapidly increase across the range of human exposures, as would be implied by the current log-linear relative risk methodology implemented in GAINS and other related models [20,34,36]. The integrated model is consistent with a biological saturation hypothesis for exposures to fine particles and the mechanisms underlying respiratory and cardiovascular disease [14,15,16].

While PM_2.5_ is regulated by mass concentration, studies indicate that the chemical composition of inhaled pollution (especially transition metals, organic compounds, semiquinones, and endotoxins) also directly influences cardiovascular risks [37]. Despite mounting toxicological evidence lending credence to the saturation hypothesis, the observed risk pattern across the range of documented human exposures to PM_2.5_ may also reflect exposure misclassification at high PM_2.5_ levels, competing mortality risks at high levels, and/or decreased inhalation rates for the heaviest of smokers that skew risk estimates downward [34]. While recent evidence implicates specific chemical components of PM_2.5_ in health risks [38], the dearth of species-specific knowledge in exposure patterns (from emissions and atmospheric modeling) and health risks (from epidemiologic studies) precludes a comprehensive analysis of the collective impact of PM_2.5_ constituents in a health impact assessment framework. This study aims to better quantify the effects of applying the integrated exposure-response functions on life expectancy in India by illuminating effects beyond average national impacts. By better spatially characterizing future risks associated with exposures to high levels of PM_2.5_ based on specific health endpoints, this work responds to calls for a more context-specific approach to health impact assessment for air pollution in India [21,22,39,40].

## 2. Materials and Methods

**Figure 1:**
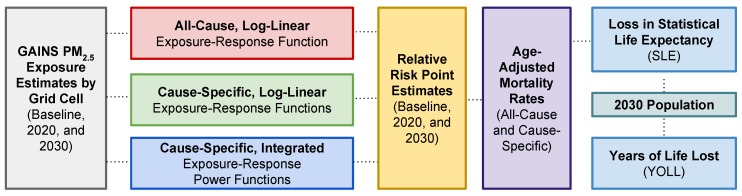
Steps in a comparative health impact analysis utilizing PM_2.5_ exposure estimates for India from the Greenhouse Gas-Air Pollution Interactions and Synergies (GAINS) model, three distinct exposure-response functions, and baseline population mortality rate estimates. Through this sequence, we estimate losses in statistical life expectancy (SLE) and years of life lost (YOLL) due to ambient exposures in 2030.

Estimates of air pollution-related health impacts are calculated using three sets of relative risk factors: the log-linear all-cause premature mortality estimate from Pope et al., (2002) [24], the log-linear cause-specific premature mortality estimate from CPS-II, and the integrated cause-specific premature mortality functions posited in the GBD assessment. These three approaches for estimating the health impacts of chronic exposure to PM_2.5_ are compared to demonstrate the range of uncertainty characteristic of health impact assessments. Specifically, as shown in Figure 1, exposure scenarios for India in GAINS are utilized to estimate the population health impact of PM_2.5_ pollution in 2030. Health impacts are estimated both in reduced statistical life expectancy (SLE) and cumulative years of life lost (YOLL). This work explores the impacts of both the newly-proposed shape of the exposure-response function as well as the disaggregation of all-cause mortality analysis into mortality estimation for specific causes of death.

### 2.1. Baseline Mortality Rates

In order to calculate changes in mortality patterns due to chronic exposure to PM_2.5_, annual cause-specific mortality rates were first calculated. WHO cause-specific mortality data for India in 2008 were used for two age groups (15–59 and 60–100) and three categories of death: lung cancer (malignant neoplasms of the trachea, bronchus, and lung), cardiovascular disease (ischemic heart disease and cerebrovascular disease), and respiratory disease (chronic obstructive pulmonary disease, and acute lower respiratory infection) [41]. Table 2 includes these values along with their population denominators.

The total number of estimated deaths for each specific cause was divided by the annual estimate of total deaths in each age group to arrive at an age-specific, cause-specific annual mortality rate. The mortality rates for the age 15–59 group were scaled to reflect the overall mortality rate for all adults above age 30 using weighting based on age-specific population counts (see Section 2.5). Age-specific mortality rates for the two age-groups were summed and weighted by the 2008 population shares of each group (30–59 year olds and 60–100 year olds) to arrive at an annual age-adjusted cause-specific proportion of the total mortality rate (Table 3). The adjusted rates were applied uniformly to the above-30 population. For the log-linear cause-specific estimates, age-adjusted mortality rates were consolidated into three categories: cardiovascular disease (ischemic heart disease and stroke), respiratory disease (chronic obstructive pulmonary disease and acute lower respiratory infection), and lung cancer.

Age-adjusted mortality shares of mortality rates for air pollution-related disease indicate that ischemic heart disease accounts for the majority of deaths, followed by acute lower respiratory infections, stroke, chronic obstructive pulmonary disease, and lung cancer.

### 2.2. Assumptions in GAINS

Health impact assessment for PM_2.5_ in GAINS assumes human exposure to PM_2.5_ from both primary sources (black carbon, organic carbon, other organic matter, and mineral dust) and secondary inorganic aerosols formed from the emissions of SO_2_, NO_X_, and NH_3_. No health impacts are quantified for exposure to PM_2.5_ stemming from natural sources and secondary organic aerosols, as it is assumed that these emissions are not as amenable to human interventions. Moreover, adverse health effects are not quantified for concentrations lower than 7 µg/m^3^; although there is not convincing evidence that a safe threshold for PM_2.5_-related health effects exists [43,44,45,46,47], this level is consistent with the GBD counterfactual concentration of 5.8–8.8 µg/m^3^ used for health impact estimates [2,47,48,49]. In urban areas, health effects are correlated with annual mean urban background levels. Health impacts and baseline mortality rates are calculated for the exposed population aged 30–100 years. This calculation assumes that all individuals will remain exposed to the exposure level calculated for the rest of their lifetimes.

#### 2.2.1. Point Estimates of Relative Risk

GAINS estimates long-term health impacts from PM_2.5_ exposure based on a single, large-scale cohort study estimating a concentration-response function for all-cause mortality [24]. Such studies utilize the Cox proportional hazards model, which relates changes in a stress variable (here, PM_2.5_ concentration) to a proportional increase in the underlying hazard (here, mortality rate) by a proportionality factor (also known as the relative risk) [50]. This model expresses the number of fatalities in a time period *Y* (usually defined as one year) as a function of baseline mortality rates (*Y*_0_), PM_2.5_ concentrations, and the relative risk factor (*β*) for an exposure (PM) in µg/m^3^ is:

*Y* = *Y*_0_ * e^β* PM^

In this model, the annual baseline death rate changes as a function of level of PM_2.5_ exposure, and the associated relative risk (RR_LOGLINEAR_) is:

*RR_LOGLINEAR_* (*PM*) = *exp*(β* PM)

**Figure 2:**
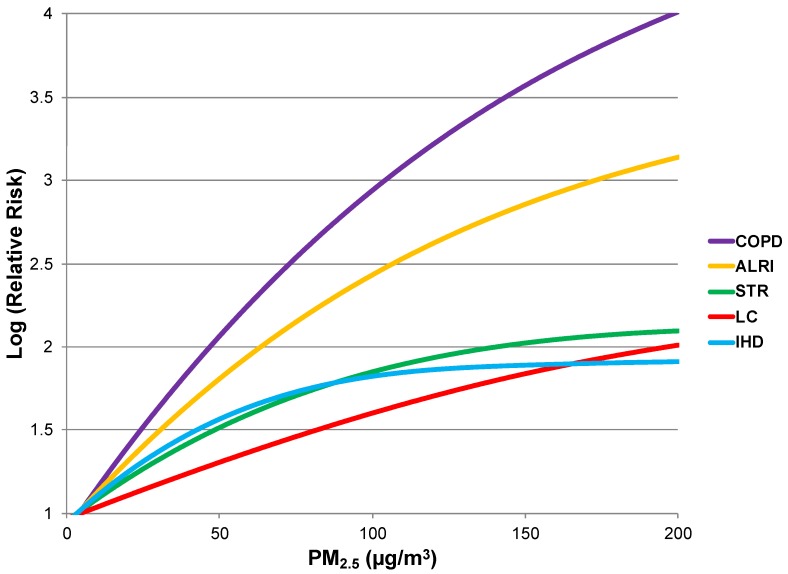
Integrated exposure-response relative risk estimates for PM_2.5_ exposure (ALRI, acute lower respiratory infection; COPD, chronic obstructive pulmonary disease; IHD, ischemic heart disease; LC, lung cancer; STR, stroke) [20]. For concentrations below 7 µg/m^3^, log-relative risk is assumed to be 1.

While *β* is small and behaves quasi-linearly in the exposure range studied in the United States (average of 17.7, SD of 3.7 µg/m^3^), the GBD assessment posited a power function for the relative risk pattern, so that risk of death increases at marginally lower rates as PM_2.5_ concentrations increase (Figure 2) [20,24]. For the power function model, risk plateaus at high exposures and the posited relative risk (RR_POWER_) is:

*for PM < PM_cf_: RR_POWER_* (*PM*) = 1

*for PM > PM_cf_: RR_POWER_* (*PM*) = 1 + *α*(1 − *exp*[−*γ*(*PM− PMcf*)*^δ^*])

where *PM_cf_* is the counterfactual concentration (7 µg/m^3^, Section 2.2) in GAINS below which no additional health risk is conferred. For this model, *γ* indicates the RR ratio comparing low to high exposure scenarios. The power of PM concentration term (*δ*) is used in order to better estimate risk over a wide range of exposures, and we apply estimates for *α*, *γ*, and *δ* from Burnett et al., (2014) [20], each derived using nonlinear regression methods.

In comparison to the all-cause mortality relative risk estimate currently deployed in GAINS, the integrated exposure-response power functions for specific causes of death are considerably steeper in slope at low exposure levels. However, the baseline mortality rate for each specific disease is by definition lower than the all-cause population mortality rate. Therefore, it is not clear whether a cause-specific health impact assessment of air pollution-related disease results in lower or higher estimates due to these compensating factors. From these models, point estimates of risk were calculated at baseline (natural background), 2020, and 2030 PM_2.5_ levels.

#### 2.2.2. Incremental Risk

Once point estimates of relative risk were determined, incremental risk slopes were calculated to linearly approximate the concentration-response power functions between the three points. Slopes were log-transformed for application in later calculations of SLE and for comparison with parameter estimates in the current literature. The general formula for the incremental risk value (*R*) is:

*R* = *Log* (*RR_1_* − *RR_2_* + 1)/ Δ*PM*

where the RR subscripts indicate point estimates of relative risk for specific causes of death for scenarios considered in pairs (baseline PM_2.5_ levels and 2020 levels, 2020 levels and 2030 levels).

#### 2.2.3. Urban and Rural Shares of Pollution

GAINS accounts for spatial heterogeneity of PM_2.5_ levels within each grid cell through its incorporation of urban shares for the year 2030. As a result, model predictions include PM_2.5_ levels for both rural and urban areas within each grid (these values coincide if the urban share is zero), enhancing geographic resolution of exposure assessment compared to past approaches. For estimates of relative risk, separate rural and urban PM_2.5_ estimates were utilized. Estimates of grid-averaged SLE were based on weighting of relative risks using the urban share fraction.

### 2.3. Scenario Selection

GAINS modeling of future PM_2.5_ levels in India is conservative because it only incorporates emissions trends but does not explicitly calculate future climate change impacts on pollution, even though climate change could exacerbate surface concentrations of this pollutant [51,52]. Global climate modeling suggests a future increase in the frequency of stagnant air masses for mid-latitudes worldwide, suggesting further accumulation of fine particles [53,54]. Moreover, changes in north-south gradients of sea surface temperatures could inhibit summer monsoon circulation and thus decrease rainfall (a key driver of particle deposition) over land [51]. In addition to direct effects of climate forcing on reduced precipitation rates, satellite data reveal that urban and industrial air pollution can suppress precipitation, as polluted plumes are comprised of many, but inefficiently small, cloud condensation nuclei [55,56]. These findings support the view that PM_2.5_ pollution will remain an important air quality threat in India even if emissions do not drastically rise in the future.

In order to estimate health impacts in 2030 in light of the integrated exposure-response functions, we used GAINS estimates of annual PM_2.5_ concentrations in each grid under baseline natural background conditions, in 2020 under a maximum feasible reduction scenario, and in 2030 under a business-as-usual emissions scenario. Point relative risk values were calculated for each of these three PM_2.5_ levels, and log-linear estimates of risk were calculated for use in incremental life expectancy calculations. Choice of the intermediate scenario for 2020 allowed us to better capture the shape of the revised exposure-response power function with log-linear slope estimates between point estimates of relative risk. In contrast to the current method, the integrated exposure-response functions display higher slopes at lower levels of PM_2.5_, and flatter slopes as high levels. The point estimate with the intermediate 2020 value also reduces error in approximating the revised curve with piecewise log-linear functions.

In grid cells with urban populations, an average annual PM_2.5_ level for the entire grid was calculated by summing the urban and rural shares and weighting the urban PM_2.5_ level by the urban fraction factor. The use of this piecewise function allowed us to capture health impacts at lower exposure levels, where the new exposure-estimates suggest that incremental health impacts are proportionally higher. Moreover, the choice of these scenarios for 2020 and 2030 represents a realistic estimate of future Indian air pollution levels and therefore a reasonable case study for health impact analysis. We also calculated SLE impacts using both log-linear, all-cause and log-linear, cause-specific models, each comparing 2030 pollution levels to baseline natural background levels. The log-linear form of these models obviates the need for 2020 pollution and risk point estimates.

### 2.4. Statistical Life Expectancy

Changes in SLE in India due to increased pollution between baseline natural background levels and 2020 and between 2020 levels and 2030 levels were calculated for each of five specific outcomes implicated in air pollution-related mortality. The basic methodology follows the approach recommended by the Task Force on Health [57]. This method uses cohort- and country-specific mortality data to estimate a survival function over time. This function is modified by exposure to PM_2.5_ pollution, in a relationship that can be simplified to losses in SLE for an individual. The calculation uses life table analysis and an approximation method for the calculation of the change in SLE [58]. These life expectancy values were weighted by their age-adjusted mortality rates, derived from WHO data [41]. Changes in SLE were calculated separately for each cause-specific outcome, and then multiplied by proportional factors from Table 3 and summed to arrive at cumulative estimates of changes in SLE for 2030.

### 2.5. Years of Life Lost

Because of spatial heterogeneity in the distribution of air pollution and human populations, it is important to assess the total burden of air pollution on society in addition to impacts on SLE. To assess the cumulative burden of PM_2.5_ air pollution on the Indian population, we calculated the YOLL in each grid [59]. We multiply the loss in SLE (expressed as fractions of years) by the total population (age 30–100) in each grid cell as currently estimated by GAINS. Population estimates for India from the International Data Base (IDB) of the U.S. Census Bureau were used to calculate a proportional factor of above-30 population in 2030 [42]. This factor (0.422) was applied to population estimates from GAINS to reflect the above-30 population total in each grid cell. In the YOLL estimate, we applied national-level age distribution data to calculate the total number of exposed individuals within each grid.

## 3. Results

Application of the three sets of exposure-response functions to GAINS-estimated PM_2.5_ concentration data led to health impact estimates for both changes in SLE impacts and cumulative YOLL in the Indian population.

### 3.1. National Health Impacts

PM_2.5_ pollution is predicted to reach an annual mean of 74 μg/m^3^ in 2030, nearly eight times the corresponding WHO air quality guideline [10]. Nationally, the average loss in SLE (aggregated from cause-specific outcomes) is 32.5 months (95% Confidence Interval: 29.7–35.2), compared to an average of 53.7 months (95% CI: 46.3–61.1) currently estimated in GAINS using the log-linear, all-cause mortality risk estimate. Table 4 compares the health impacts estimated by the single all-cause and two cause-specific exposure-response functions.

### 3.2. Regional Health Impacts

**Figure 3:**
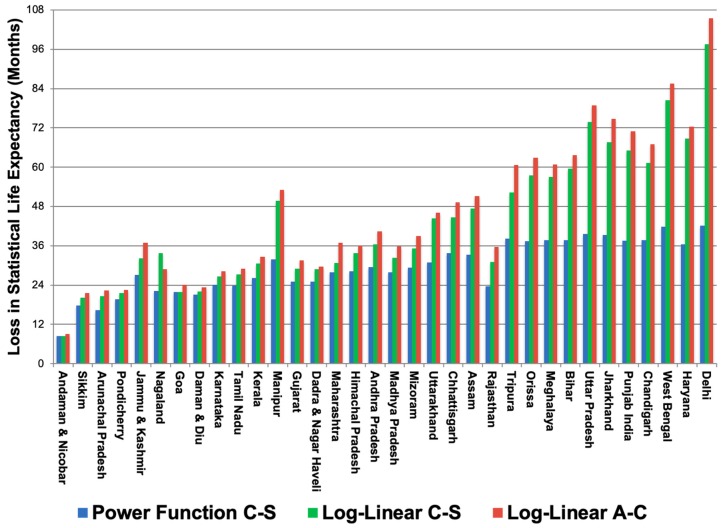
Months of SLE lost due to chronic exposure to PM_2.5_ based on three exposure-response functions: Power Function C-S (cause-specific, blue), Log-Linear C-S (cause-specific, green), and Log-Linear A-C (all-cause, red). Regions are sequenced from lowest to highest PM_2.5_ level in 2030.

**Figure 4:**
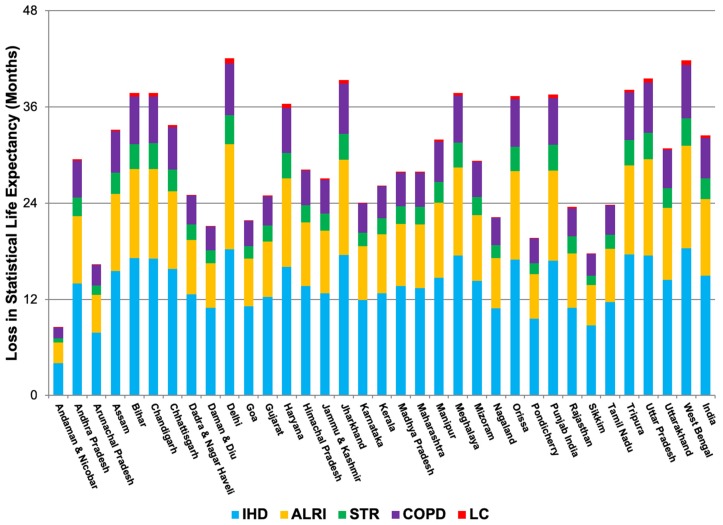
Months of SLE lost in GAINS regions due to 2030 PM_2.5_ exposures, applying cause-specific integrated exposure-response power functions (IHD, ischemic heart disease; ALRI, acute lower respiratory infection; STR, stroke; COPD, chronic obstructive pulmonary disease; LC, lung cancer).

The different SLE impacts estimated by the three models are depicted for each GAINS region in Figure 3. Regions are sequenced from lowest to highest PM_2.5_ level in 2030. Figure 4 displays the aggregation of cause-specific SLE impacts using the power function.

### 3.3. Grid Comparison

**Figure 5:**
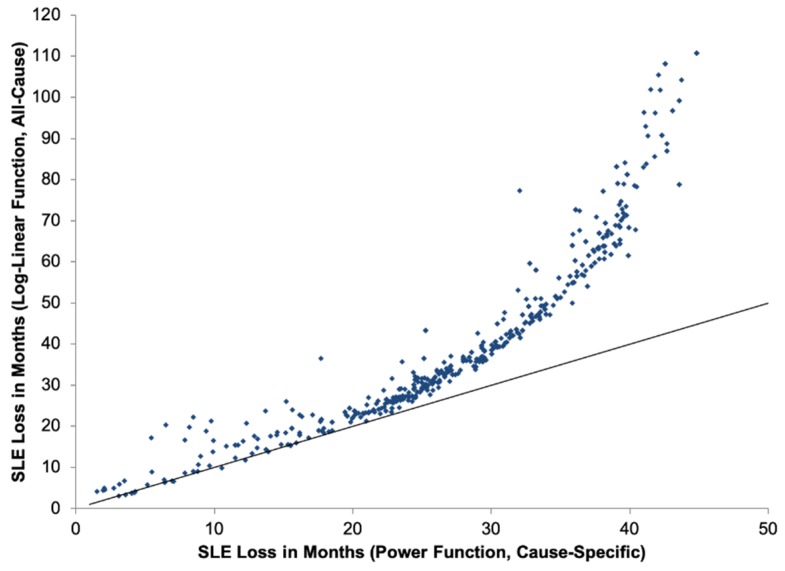
Comparison of loss in statistical life expectancy (SLE, in months) estimated for the year 2030 due to PM_2.5_ pollution in each of 553 1° GAINS grid cells, applying the cause-specific power function (horizontal axis) and all-cause log-linear function (vertical axis) exposure-response functions. The 45° line of parity is also indicated.

Figure 5 displays the contrast in SLE estimates for each grid cell in GAINS between the integrated exposure-response power function and the log-linear function. For the same level of 2030 pollution, SLE impacts peak at a much lower level using the power function.

### 3.4. Years of Life Lost

Table 5 displays the YOLL results for each GAINS region, applying both the power and log-linear functions.

## 4. Discussion

### 4.1. Three Exposure-Response Models

Three models for impacts of PM_2.5_ on health (reduced life expectancy) are represented in Figure 3. These three models (log-linear all-cause, log-linear cause-specific, and power function cause-specific) represent increasingly precise estimates of the cumulative health impacts of PM_2.5_ pollution. Results from these exposure-response functions align most in areas with relatively low levels of pollution (generally in southern India, as other studies have found, [8]). In the least polluted areas, the difference between the lowest and highest SLE estimates (log-linear cause-specific and log-linear all-cause, respectively) is 0.60 months (in the Andaban and Nicobar islands). However, in areas with poorer air quality, model estimates diverge considerably, with a difference of 63.4 months between the lowest and highest SLE estimates in Delhi (power function cause-specific and log-linear all-cause, respectively). Overall, the central estimate of 32.5 months (2.7 years) of SLE loss in 2030 is consistent with other recent national analyses of present-day air pollution impacts, which have estimated SLE losses ranging from 1.5 years to 5.3 years [8,60,61,62]. While some of these studies also considered indoor air pollution and ozone (O_3_) exposures, our finding is comparable to a PM_2.5_-only life expectancy impact of 3.2 years as calculated by Greenstone et al. [63]. 

### 4.2. Incremental Impacts

In assessing the consequences of applying revised exposure-response functions, it is important to distinguish between incremental and cumulative health impacts. The posited power function implies that the risk of death is not constant along the continuum of exposure to PM_2.5_. As a result, adverse health impacts rapidly accrue at the lower end of the exposure spectrum, but increase more slowly as one proceeds to higher levels of exposure. For example, for chronic obstructive pulmonary disease, the log-relative risk approximately triples between 10 µg/m^3^ and 20 µg/m^3^, but only increases by 7% between ambient concentrations of 100 µg/m^3^ and 110 µg/m^3^. Therefore, it is essential to consider the baseline air quality situation in assessing health benefits that might be achieved from reducing air pollution. The most striking consequences of the revised functions are seen in estimates of these incremental impacts. Given these patterns and future exposure risks [64], it is apparent that, in order to achieve the biggest health gains, ambitious actions are needed to significantly reduce the overall magnitude of ambient PM_2.5_ air pollution in India.

### 4.3. Statistical Life Expectancy

The cause-specific analysis results in health impacts that are on the same order of magnitude but generally lower than estimates using the old methodology (see Figure 3). Regional SLE impacts across all causes and ages ranged from 8.5–42.1 months, with a population-weighted mean of 32.5 months (standard deviation of 8.2 months). The regions in Figure 3 are sequenced by 2030 PM_2.5_ level; in the most polluted regions, the impacts on SLE are (as expected) highest. It is also in these places where the loss in SLE due to lung cancer is highest, accounting for 0.6 months of the total loss in SLE in Delhi.

There are, however, interesting exceptions to the general trend of increasing health effects by 2030 pollution level. The clearest example is in Rajasthan, where the 2030 PM_2.5_ level is 74.4 µg/m^3^ and cumulative SLE loss is estimated at 23.6 months. While Assam and Tripura are expected to experience similar pollution levels in 2030 (68.9 and 77.7 µg/m^3^, respectively), the latter regions are anticipated to suffer higher SLE losses (33.2 and 38.2 months, respectively) than Rajasthan. This contrast is due to differences in modeled pollution changes between the baseline natural background concentrations and 2030 business-as-usual scenario. Relative to baseline background levels, PM_2.5_ levels in Assam increased by 67.5 µg/m^3^ and Tripura by 75.6 µg/m^3^, while Rajasthan only increased 45.7 µg/m^3^. Because natural background pollution is high to begin with in Rajasthan, health impacts due to anthropogenic emissions are relatively lower. The results in Figure 3 demonstrate that the divergence between the new exposure-response methodology and current GAINS approach is greatest in areas with high levels of PM_2.5_ pollution. In Delhi, for example, the revised estimate for SLE impacts is 40% of the current GAINS estimate. By comparison, in the least polluted region (Andaman and Nicobar), the power function estimate is 95% of the SLE impact projected using current GAINS methodology.

### 4.4. Years of Life Lost

Cumulative impacts of population exposures to air pollution are helpful in conveying the total burden of human-driven PM_2.5_ pollution to policymakers. The YOLL estimate (Table 5) places the statistical life expectancy calculation in a human context, taking into account spatial heterogeneity in population density. Just as the cause-specific life expectancy results are lower than under current GAINS assumptions, when population estimates are applied to calculate cumulative Years of Life Lost (YOLL), the health impacts are diminished. Amongst regions, Andaman and Nicobar had the lowest YOLL toll (71,041) due to its small population and relatively modest PM_2.5_ levels. Uttar Pradesh had the highest YOLL burden (225,900,339) and Delhi, the location with the highest loss in SLE, accumulated 17,487,000 YOLL by 2030. Overall, 1.1 billion YOLL can be attributed to long-term exposure to ambient PM_2.5,_ or roughly one year for each person in India (including those under age 30).

### 4.5. Sensitivity Analysis

To place revised health impact estimates in the context of current GAINS calculations, a sensitivity analysis was conducted by comparing exposure-response methodology. Overall, the revised method results in consistently lower estimates in India (67% ratio of new method to old) of losses in SLE due to ambient concentrations of PM_2.5_. Nevertheless, application of the power function in less polluted settings could result in SLE estimates that are larger than those calculated here. Nationally, the revised method projects that chronic exposure to human-driven PM_2.5_ pollution reduces the average Indian citizen’s life by about 32.5 months (Table 4, Figure 3 and Figure 4). Of this total, the largest contributor to premature mortality is ischemic heart disease (15.0 months, 46.1% of total average life-shortening), followed by acute lower respiratory infection (9.5 months, 29.3%), stroke (5.1 months, 15.5%), chronic obstructive pulmonary disease (2.6 months, 8.1%), and lung cancer (0.3 months, 0.01%). The log-linear method estimated a national loss in life expectancy in 2030 at 53.7 months. However, the national picture obscures important regional variation, reflected in the relatively large standard deviation of the estimate. At the extremes, the integrated exposure-response method results in a 60% lower health impact estimate in Delhi, and a 5% lower estimate in Andaman and Nicobar. However, alternate estimates utilized in the sensitivity analysis reflect the largest population health estimate included in GAINS, utilizing the highest reported relative risk estimate from Pope et al. (2002) [24].

### 4.6. Trends in Urbanization and Population Growth

The rapid pace of economic growth on the Indian subcontinent suggests that air quality issues and their public health impacts will remain central over coming years due to increasing emissions, especially if fossil fuels remain a large portion of India’s energy portfolio [4,52]. The Indian government has championed a goal of maintaining annual economic growth above 8% for the next two decades, which would increase per-capita income by a factor of five and stimulate an increasing demand for energy [4]. India’s population is increasingly urban, and patterns of air pollution exposure are expected to shift away from indoor air towards problems of ambient PM and O_3_ in cities [10]. As a result, fine particulate matter pollution may worsen in coming decades due to continuing urban expansion and India’s reliance on coal for electricity generation [65]. By 2030, high levels of PM_2.5_ are projected to pervade across most of the country, with concentrations in the Ganges Valley increasing to more than 150 µg/m^3^ [4].

Regional variation in the concentration of PM_2.5_, combined with an increasingly urbanized Indian population, results in important geographic disparities in health. In 2030, for example, life-shortening in the city of Delhi is estimated at 108 months, in comparison to about 10 months in the Andaman and Nicobar islands [4]. The impact of lower levels of air pollution on life expectancy in India is consistent with patterns in the United States between 2000–2007: in one study, a decrease of 10 μg/m^3^ in the concentration of PM_2.5_ was associated with an increase in life expectancy of about four months [48].

### 4.7. Limitations: Model Assumptions and External Validity

The spatial resolution of the GAINS model is a key shortcoming, as combination of 1° population and exposure estimates with national incidence data provide a limited degree of spatial specificity. While GAINS provides robust national estimates of air pollution exposures, improvement in the spatial coverage and resolution of ambient air quality and baseline health incidence data is vital for estimating human exposures and health impacts more accurately [66,67]. While restricting the health impact analysis to the age 30+ group is consistent with other studies of this kind [68,69,70,71] and GAINS is not equipped to consistently apply age-specific relative risk functions for all health endpoints, this simplification biases health impact estimates.

The national-level age-specific population distributions from data extrapolated by the U.S. Census Bureau are useful for describing the country-level impacts of air pollution on life expectancy, but not for spatially-explicit estimates of the future disease burden and intervention opportunities for specific age groups. As an example of this limitation, our broad application of the health impacts of ALRI (Table 2 and Table 3) does not reflect the disproportionate burden of this category of infections on the very young (0–5 years), including a higher burden of YOLL in this age subgroup. This limitation of our study is complicated by the fact that we did not apply exposure-response analysis to other diseases like asthma and pneumonia that also contribute to premature mortality from PM_2.5_, with important age-specific effects [72,73]. Nevertheless, the relative proportion of ALRI-associated impacts is consistent with a city-specific analysis in Varanasi, India [74]. Stratification of health estimates by age group and consideration of a wider range of impacts for the entire population would allow for a more complete understanding of the cumulative impacts of national PM_2.5_ exposures.

Moreover, this study does not apply the integrated exposure-response functions to estimates of indoor air pollution. Solid fuel combustion is still widespread in India, and a large share of health impacts from outdoor exposure to PM_2.5_ has been linked to this source [4,75]. Future research efforts using GAINS could more fully consider the health impacts of both indoor and outdoor air pollution to better quantify the burden for both children and adults. The integrated exposure-response function may be deployed in health impact analyses of indoor air pollution exposures, although the exposure patterns for women and children may be distinct from those for ambient air pollution.

In addition to causing premature mortality, the morbidity impacts of PM_2.5_ pollution are well-documented [76]. These effects include irregular heartbeat, aggravated asthma, airway irritation, and decreased lung function. None of these impacts are addressed under current GAINS methodology despite their contribution to ill health and reduced human productivity. Moreover, exposure to PM_2.5_ is known to cause adverse health impacts on both acute and chronic time scales. In this work, acute impacts are assumed to be embedded in annual mortality estimates, but air quality and health intervention planning would benefit from a quantitative understanding of the temporal dynamics of health effects.

Calculations for the total losses in SLE attribute health impacts to PM_2.5_ sourced from anthropogenic activities, but background levels of this pollutant within the model are already at dangerous levels at several places in Northwest India. Moreover, evidence indicates no safe threshold for chronic PM_2.5_ exposure below which no adverse health impacts (e.g., a relative risk ≤1) would be expected [47,48], so total population health impacts quantified here are conservative estimates.

Although the GAINS model considers urban and rural concentrations of PM_2.5_, urban fractions are held constant in this study between baseline conditions and the future. In reality, the urban population is expected to grow by an estimated 400 million between now and 2050 to more than 700 million in total [77]. As a result, changing patterns of urbanization in the country are not adequately captured, biasing health impact results downward [78]. It is important to recognize that because outdoor air pollution-related mortality was the health outcome of interest in this study, estimates are only for the population aged 30–100. India still growing quickly and has a large youth population. The total fertility rate in 2003 ranged between 2.2 children per woman in urban areas to 3.2 in rural settings, with a national average of 3.0 [79]. In coming years, a surge in the above-30 population is anticipated [42]. The above-30 population estimates applied in this study use current GAINS population totals for ease of comparability; in truth, future health impacts will be larger due to population growth. Moreover, none of the morbidity impacts of PM_2.5_ are addressed under current GAINS methodology despite their contribution to ill health and reduced human productivity.

In considering the results of this modeling, we also acknowledge the overall health burden in South Asia and some unique risks to its population. A 2007 study found that people native to India, Pakistan, Bangladesh, Nepal and Sri Lanka typically die from heart disease five to ten years earlier than those from other ethnic groups [80]. Moreover, there is increasing evidence that traditional risk factors for acute myocardial infarction (including obesity, high blood pressure, elevated cholesterol levels, and diabetes) attack the cardiovascular system more aggressively in this population. These findings, and those of related studies on lung function and underlying environmental health determinants ([81,82]), suggest that the application of cohort study results estimating premature mortality from cardiovascular disease due to PM_2.5_ exposure may underestimate the health risk for particular populations in South Asia.

### 4.8. Policy Implications

The integrated exposure-response functions have important implications for air quality management and public health policy. Under the assumption of biological saturation and a plateau of health risk, areas with more polluted air at baseline are less amenable to improvements in health relative to their cleaner counterparts, because marginal health impacts are highest at relatively low levels of PM_2.5_ [83]. In contrast to the current log-linear, all-cause mortality risk function, the new methods indicate that once a threshold of PM_2.5_ has been passed, health impacts accrue at a declining rate (Figure 5). As a result, in areas experiencing high levels of pollution, only substantial improvements in air quality will produce desired dividends in improvements to population health. Nevertheless, application of the integrated exposure-response functions estimated a global total of 3.2 million premature deaths and identified of outdoor air pollution as the ninth leading risk factor for disease and early death in 2010 [2].

More broadly, trends in air pollution and associated health impacts are only a component of the entire burden of disease in India. As the country continues to progress through the epidemiologic transition, the health sector is tasked with addressing threats to well-being from a number of sources. Better estimates of the long-term impact of PM_2.5_ exposure will allow for improved public health planning, and could motivate policies to further improve air quality. It is unclear how helpful the quantification of specific-cause mortality risks is in the absence of baseline incidence data at a more local level. Moreover, in communicating health impact assessments to policymakers, premature mortality estimates linked to air pollution should be put in adequate context. This study did not consider lag effects in relating health outcomes to pollutant exposures, as long-term exposures are related to average impacts on SLE.

In addition to cause-specific mortality estimates, recent studies posit age-specific exposure-response functions for ischemic heart disease as a function of chronic exposure to fine particulate matter [20]. Incorporation of age-specific exposure-response functions was outside of the scope of this study, but would be useful in determining the proportional impacts of cause-specific mortality from air pollution exposures. Because ischemic heart disease comprises such a large percentage of air pollution-related disease, application of these new functions could have important implications for population health impact assessments in this region.

## 5. Conclusions

We applied integrated exposure-response functions to estimate the cause-specific mortality risks associated with ambient PM_2.5_ exposures in India in 2030 using Greenhouse Gas-Air Pollution Interactions and Synergies (GAINS) model projections. Losses in statistical life expectancy (SLE) were calculated based on population-weighted exposure estimates, relative risk estimates for mortality (all-cause and cause-specific), and baseline national mortality rates. Calculated reductions in SLE were aggregated and weighted using national age-adjusted, cause-specific mortality rates. In our modeling, 2030 PM_2.5_ pollution in India reached an annual mean of 74 μg/m^3^, nearly eight times the corresponding World Health Organization air quality guideline. The average loss in SLE was 32.5 months (95% Confidence Interval: 29.7–35.2, regional range: 8.5–42.0), compared to an average of 53.7 months (95% CI: 46.3–61.1) using methods currently applied in GAINS and similar models. Although these impacts are large, our transparent methods likely underestimate the total health burden caused by PM_2.5_ exposures due to model assumptions on minimum age thresholds of pollution effects and a limited subset of health endpoints analyzed. Application of the revised exposure-response models suggest that the most polluted areas in India will reap major health benefits only with substantial improvements in air quality.

## Figures and Tables

**Table 1:** Relative risk factors for specific causes of death for a 1 μg/m^3^ change in PM_2.5_ exposure from the American Cancer Society Cancer Prevention Study-II [3,24].

*Cause of Death*	*Relative Risk* *(Central Estimate)*	95% Confidence Interval
All-Cause	1.006	1.002–1.100
Cardiovascular Disease (Ischemic Heart Disease and Stroke)	1.017	1.011–1.024
Respiratory Disease (Chronic Obstructive Pulmonary Disease and Acute Lower Respiratory Infection)	1.006	0.097–1.016
Lung Cancer	1.014	1.006–1.023

**Table 2:** Estimated deaths in India (2008) by cause [41]. Age-specific 2008 population estimates for India from the International Data Base (IDB) of the U.S. Census Bureau [42]. ^*^ Population estimate for population aged 30–59, used in age-adjusted mortality rates for population aged 30–100 (Table 3).

Age Group	Cause of Premature Mortality	Mortality Estimate	Total Deaths	Population
15–59	Ischemic Heart Disease	672,000	3,092,700	720,694,000 (391,440,200 *)
	Stroke	180,200		
	Acute Lower Respiratory Illness	214,400		
	Chronic Obstructive Pulmonary Disease	44,900		
	Lung Cancer	20,600		
60–100	Ischemic Heart Disease	826,000	4,678,400	90,312,849
	Stroke	646,400		
	Acute Lower Respiratory Illness	870,300		
	Chronic Obstructive Pulmonary Disease	220,000		
	Lung Cancer	31,500		

**Table 3:** Annual age-adjusted proportion of mortality rate for India in 2008 for adults aged 30–100 in 2008.

Cause of Premature Mortality	Age-Adjusted Share of Annual Mortality Rate (%)
Ischemic Heart Disease	21.0
Acute Lower Respiratory Illness	9.1
Stroke	7.3
Chronic Obstructive Pulmonary Disease	2.1
Lung Cancer	0.7

**Table 4:** Changes in statistical life expectancy (SLE) using log-linear= all-cause, log-linear cause-specific, and power function cause-specific exposure-response functions for premature mortality and GAINS PM_2.5_ exposure estimates. National averages calculated from population-based weighting of individual grids.

Exposure-Response Function	Average National Loss in SLE (Months)	95% Confidence Interval (Months)
Log-Linear, All-Cause	53.7	46.3–61.1
Log-Linear, Cause-Specific	49.4	42.7–56.1
Power Function, Cause-Specific	32.5	29.7–35.2

**Table 5:** Years of Life Lost (YOLL) estimated for the year 2030 due to PM_2.5_ pollution in India using integrated, cause-specific exposure-response functions compared to the current (log-linear, all-cause) GAINS approach. Regional averages calculated from population-based weighting of individual grids.

Region	Population Age 30–100	YOLL (Power Function, Cause-Specific Mortality)	YOLL (Log-Linear, All-Cause Mortality)
Andaman & Nicobar	100,330	71,041	75,405
Andhra Pradesh	31,334,161	76,806,481	105,513,079
Arunachal Pradesh	626,257	852,835	1,168,634
Assam	10,700,667	29,603,288	45,577,660
Bihar	33,518,253	105,402,006	177,643,919
Chandigarh	188,466	593,341	1,052,555
Chhattisgarh	8,681,291	24,396,711	35,680,365
Dadra & N. Haveli	48,125	100,420	118,675
Daman & Diu	48,744	85,837	94,496
Delhi	4,988,542	17,486,999	43,833,733
Goa	528,426	962,794	1,061,004
Gujarat	20,791,047	43,259,945	54,757,350
Haryana	9,389,899	28,457,447	56,689,670
Himachal Pradesh	2,604,428	6,119,326	7,806,830
Jammu & Kashmir	4,047,044	9,127,280	12,470,991
Jharkhand	11,598,975	38,033,918	72,210,222
Karnataka	21,664,583	43,394,208	50,991,253
Kerala	13,238,535	28,926,694	36,050,459
Madhya Pradesh	24,900,682	58,026,813	74,456,342
Maharashtra	38,349,724	89,336,917	118,150,353
Manipur	954,847	2,538,910	4,224,297
Meghalaya	1,176,235	3,702,158	5,955,767
Mizoram	359,130	877,077	1,168,562
Nagaland	717,811	1,330,132	1,721,418
Orissa	15,167,417	47,199,759	79,501,011
Pondicherry	55,121	90,030	103,332
Punjab	10,172,018	31,863,412	60,150,281
Rajasthan	23,317,953	45,789,791	69,256,961
Sikkim	241,827	356,728	435,056
Tamil Nadu	25,184,943	49,909,735	60,974,345
Tripura	1,314,249	4,179,375	6,652,071
Uttar Pradesh	68,524,470	225,900,339	450,382,887
Uttarakhand	3,574,632	9,192,348	13,715,040
West Bengal	32,806,548	114,273,583	233,985,679
**National Total**	**420,915,379**	**1,106,384,265**	**1,883,629,702**

## References

[1] World Health Organization (2001). Quantification of Health Effects of Exposure to Air Pollution.

[2] Lim S.S., Vos T., Flaxman A.D., Danaei G., Shibuya K., Adair-Rohani H., AlMazroa M.A., Amann M., Anderson H.R., Andrews K.G. (2012). A comparative risk assessment of burden of disease and injury attributable to 67 risk factors and risk factor clusters in 21 regions, 1990–2010: A systematic analysis for the Global Burden of Disease Study 2010. Lancet.

[3] Cohen A.J., Anderson H.R., Ostro B., Pandey K.D., Krzyzanowski M., Künzli N., Gutschmidt K., Pope III C., Romieu I., Samet J.M. (2004). Urban air pollution. Comp. Quantif. Health Risks Glob. Reg. Burd. Dis. Due Sel. Major Risk Factors.

[4] Purohit P., Amann M., Mathur R., Gupta I., Marwah S., Verma V., Bertok I., Borken-Kleefeld J., Chambers A., Cofala J. (2010). GAINS-Asia. Scenarios for Cost-Effective Control of Air Pollution and Greenhouse Gases in India.

[5] Chowdhury Z., Zheng M., Schauer J.J., Sheesley R.J., Salmon L.G., Cass G.R., Russell A.G. (2007). Speciation of ambient fine organic carbon particles and source apportionment of PM_2.5_ in Indian cities. J. Geophys. Res. Atmos..

[6] Bruce N., Perez-Padilla R., Albalak R. (2000). Indoor air pollution in developing countries: A major environmental and public health challenge. Bull. World Health Organ..

[7] Smith K. (2000). National Burden of Disease in India from Indoor Air Pollution. Proc. Natl. Acad. Sci. USA.

[8] Collaborators I.S.-L.D.B.I.A.P. (2018). The impact of Air Pollution on Deaths, Disease Burden, and Life Expectancy Across the States of India: The Global Burden of Disease Study 2017. Lancet Planet Health.

[9] Brauer M., Amann M., Burnett R.T., Cohen A., Dentener F., Ezzati M., Henderson S.B., Krzyzanowski M., Martin R.V., Van Dingenen R. (2012). Exposure Assessment for Estimation of the Global Burden of Disease Attributable to Outdoor Air Pollution. Environ. Sci. Technol..

[10] World Health Organization (2006). Air Quality Guidelines: Global Update 2005: Particulate Matter, Ozone, Nitrogen Dioxide, and Sulfur Dioxide.

[11] Tonne C., Salmon M., Sanchez M., Sreekanth V., Bhogadi S., Sambandam S., Balakrishnan K., Kinra S., Marshall J.D. (2017). Integrated assessment of exposure to PM_2.5_ in South India and its relation with cardiovascular risk: Design of the CHAI observational cohort study. Int. J. Hyg. Environ. Health.

[12] Amann M., Bertok I., Borken J., Chambers A., Cofala J., Dentener F., Heyes C., Hoglund L., Klimont Z., Purohit P. (2008). GAINS-Asia: A tool to combat air pollution and climate change simultaneously; Methodology.

[13] Hoek G., Krishnan R.M., Beelen R., Peters A., Ostro B., Brunekreef B., Kaufman J.D. (2013). Long-term air pollution exposure and cardio-respiratory mortality: A review. Environ. Health.

[14] Seaton A., Godden D., MacNee W., Donaldson K. (1995). Particulate air pollution and acute health effects. Lancet.

[15] Ambrose J.A., Barua R.S. (2004). The pathophysiology of cigarette smoking and cardiovascular disease. J. Am. Coll. Cardiol..

[16] Strulovici-Barel Y., Omberg L., O’Mahony M., Gordon C., Hollmann C., Tilley A.E., Salit J., Mezey J., Harvey B.-G., Crystal R.G. (2010). Threshold of Biologic Responses of the Small Airway Epithelium to Low Levels of Tobacco Smoke. Am. J. Respir. Crit. Care Med..

[17] Smith K.R., Mehta S. (2003). The burden of disease from indoor air pollution in developing countries: Comparison of estimates. Int. J. Hyg. Environ. Health.

[18] Malmqvist E., Oudin A., Pascal M., Medina S. (2018). Choices Behind Numbers: A Review of the Major Air Pollution Health Impact Assessments in Europe. Curr. Environ. Health Rep..

[19] Brunekreef B. (1997). Air pollution and life expectancy: Is there a relation?. Occup. Environ. Med..

[20] Burnett R.T., Pope C.A., Ezzati M., Olives C., Lim S.S., Mehta S., Shin H.H., Singh G., Hubbell B., Brauer M. (2014). An Integrated Risk Function for Estimating the Global Burden of Disease Attributable to Ambient Fine Particulate Matter Exposure. Environ. Health Perspect..

[21] Ministry of Environment, Forests, and Climate Change (MOEFCC) Draft National Clean Air Plan. http://envfor.nic.in/sites/default/files/NCAP%20with%20annex-ilovepdf-compressed.pdf.

[22] Gordon T., Balakrishnan K., Dey S., Rajagopalan S., Thornburg J., Thurston G., Agrawal A., Collman G., Guleria R., Limaye S. (2018). Air pollution health research priorities for India: Perspectives of the Indo-U.S. Communities of Researchers. Environ. Int..

[23] Cohen A.J., Ross Anderson H., Ostro B., Pandey K.D., Krzyzanowski M., Künzli N., Gutschmidt K., Pope A., Romieu I., Samet J.M. (2005). The Global Burden of Disease Due to Outdoor Air Pollution. J. Toxicol. Environ. Health A.

[24] Pope III C.A., Burnett R.T., Thun M.J., Calle E.E., Krewski D., Ito K., Thurston G.D. (2002). Lung cancer, cardiopulmonary mortality, and long-term exposure to fine particulate air pollution. J. Am. Med. Assoc..

[25] Walter A. (2004). Time-Series Analysis of Air Pollution and Mortality: A Statistical Review.

[26] Smith K.R., Frumkin H., Balakrishnan K., Butler C.D., Chafe Z.A., Fairlie I., Kinney P., Kjellstrom T., Mauzerall D.L., McKone T.E. (2013). Energy and Human Health. Annu. Rev. Public Health.

[27] Central Pollution Control Board National Ambient Air Quality Status & Trends in India. http://cpcb.nic.in/openpdffile.php?id=UmVwb3J0RmlsZXMvMzJfMTQ1ODEyNjU5MV9OZXdJdGVtXzE5Ml9OQUFRU1RJLnBkZg.

[28] Limaye V.S., Knowlton K., Sarkar S., Ganguly P.S., Pingle S., Dutta P., M L.S., Tiwari A., Solanki B., Shah C. (2018). Development of Ahmedabad’s Air Information and Response (AIR) Plan to Protect Public Health. Int. J. Environ. Res. Public Health.

[29] Dey S., Di Girolamo L., van Donkelaar A., Tripathi S., Gupta T., Mohan M. (2012). Variability of outdoor fine particulate (PM_2.5_) concentration in the Indian subcontinent: A remote sensing approach. Remote Sens. Environ..

[30] Chowdhury S., Dey S. (2016). Cause-specific premature death from ambient PM_2.5_ exposure in India: Estimate adjusted for baseline mortality. Environ. Int..

[31] Kumar N., Chu A., Foster A. (2007). An empirical relationship between PM_2.5_ and aerosol optical depth in Delhi Metropolitan. Atmos. Environ..

[32] Central Pollution Control Board National Ambient Air Quality Standards. http://cpcb.nic.in/air-quality-standard/.

[33] Apte J.S., Marshall J.D., Cohen A.J., Brauer M. (2015). Addressing Global Mortality from Ambient PM_2.5_. Environ. Sci. Technol..

[34] Pope III C.A., Burnett R.T., Turner M.C., Cohen A., Krewski D., Jerrett M., Gapstur S.M., Thun M.J. (2011). Lung Cancer and Cardiovascular Disease Mortality Associated with Ambient Air Pollution and Cigarette Smoke: Shape of the Exposure–Response Relationships. Environ. Health Perspect..

[35] McCracken J.P., Wellenius G.A., Bloomfield G.S., Brook R.D., Tolunay H.E., Dockery D.W., Rabadan-Diehl C., Checkley W., Rajagopalan S. (2012). Household Air Pollution from Solid Fuel Use. Glob. Heart.

[36] US Environmental Protection Agency (2010). Environmental Benefits Mapping and Analysis Program (BenMAP) 4.0.

[37] Brook R.D., Rajagopalan S., Pope C.A., Brook J.R., Bhatnagar A., Diez-Roux A.V., Holguin F., Hong Y., Luepker R.V., Mittleman M.A. (2010). Particulate Matter Air Pollution and Cardiovascular Disease: An Update to the Scientific Statement from the American Heart Association. Circulation.

[38] Wu S., Deng F., Wang X., Wei H., Shima M., Huang J., Lv H., Hao Y., Zheng C., Qin Y. (2013). Association of lung function in a panel of young healthy adults with various chemical components of ambient fine particulate air pollution in Beijing, China. Atmos. Environ..

[39] Ministry of Health and Family Welfare (2015). Report of the Steering Committee on Air Pollution and Health-Related Issues.

[40] Pant P., Guttikunda S.K., Peltier R.E. (2016). Exposure to particulate matter in India: A synthesis of findings and future directions. Environ. Res..

[41] World Health Organization, World Health Organization (2009). Global Health Risks: Mortality and Burden of Disease Attributable to Selected Major Risks.

[42] U.S. Census Bureau International Programs—Information Gateway—U.S. Census Bureau. http://www.census.gov/population/international/data/idb/informationGateway.php.

[43] Crouse D.L., Peters P.A., van Donkelaar A., Goldberg M.S., Villeneuve P.J., Brion O., Khan S., Atari D.O., Jerrett M., Pope C.A. (2012). Risk of Nonaccidental and Cardiovascular Mortality in Relation to Long-term Exposure to Low Concentrations of Fine Particulate Matter: A Canadian National-Level Cohort Study. Environ. Health Perspect..

[44] Di Q., Wang Y., Zanobetti A., Wang Y., Koutrakis P., Choirat C., Dominici F., Schwartz J.D. (2017). Air Pollution and Mortality in the Medicare Population. N. Engl. J. Med..

[45] Fann N., Baker K.R., Chan E.A.W., Eyth A., Macpherson A., Miller E., Snyder J. (2018). Assessing Human Health PM_2.5_ and Ozone Impacts from U.S. Oil and Natural Gas Sector Emissions in 2025. Environ. Sci. Technol..

[46] Schwartz J.D., Wang Y., Kloog I., Yitshak-Sade M., Dominici F., Zanobetti A. (2018). Estimating the Effects of PM_2.5_ on Life Expectancy Using Causal Modeling Methods. Environ. Health Perspect..

[47] U.S. Environmental Protection Agency, Office of Air Quality Planning and Standards Health and Environmental Impact Division, Air Benefit-Cost Group Summary of Expert Opinions on the Existence of a Threshold in the Concentration-Response Function for PM_2.5_-related Mortality. http://www.epa.gov/ttn/ecas/regdata/Benefits/thresholdstsd.pdf.

[48] Correia A.W., Pope C.A., Dockery D.W., Wang Y., Ezzati M., Dominici F. (2013). Effect of Air Pollution Control on Life Expectancy in the United States. Epidemiology.

[49] Shin H.H., Cohen A.J., Pope C.A., Ezzati M., Lim S.S., Hubbell B.J., Burnett R.T. (2016). Meta-analysis methods to estimate the shape and uncertainty in the association between long-term exposure to ambient fine particulate matter and cause-specific mortality over the global concentration range. Risk Anal..

[50] Cox D.R. (1972). Regression Models and Life-Tables. J. R. Stat. Soc..

[51] Ramanathan V., Feng Y. (2009). Air pollution, greenhouse gases and climate change: Global and regional perspectives. Atmos. Environ..

[52] Pommier M., Fagerli H., Gauss M., Simpson D., Sharma S., Sinha V., Ghude S.D., Landgren O., Nyiri A., Wind P. (2018). Impact of regional climate change and future emission scenarios on surface O_3_ and PM_2.5_ over India. Atmos. Chem. Phys..

[53] Mickley L.J., Jacob D.J., Field B.D., Rind D. (2004). Effects of future climate change on regional air pollution episodes in the United States. Geophys. Res. Lett..

[54] Jacob D.J., Winner D.A. (2009). Effect of climate change on air quality. Atmos. Environ..

[55] Rosenfeld D. (2000). Suppression of Rain and Snow by Urban and Industrial Air Pollution. Science.

[56] Givati A., Rosenfeld D. (2004). Quantifying precipitation suppression due to air pollution. J. Appl. Meteorol..

[57] Joint Task Force on the Health, Aspects of Air Pollution, Executive Body for the Convention on Long-range Transboundary Air Pollution (2004). Modelling and Assessment of the Health Impact of Particulate Matter and Ozone.

[58] Vaupel J.W., Yashin A.I. (1985). Heterogeneity’s Ruses: Some Surprising Effects of Selection on Population Dynamics. Am. Stat..

[59] World Health Organization Years of Life Lost (Percentage of Total). https://www.who.int/whosis/whostat2006YearsOfLifeLost.pdf.

[60] Apte J.S., Brauer M., Cohen A.J., Ezzati M., Pope C.A. (2018). Ambient PM_2.5_ Reduces Global and Regional Life Expectancy. Environ. Sci. Technol. Lett..

[61] Berkowitz B., Muyskens J., Sharma M., Ulmanu M. How Many Years Do We Lose to the Air We Breathe?. https://www.washingtonpost.com/graphics/2018/national/health-science/lost-years/.

[62] Ghude S.D., Chate D., Jena C., Beig G., Kumar R., Barth M., Pfister G., Fadnavis S., Pithani P. (2016). Premature mortality in India due to PM_2.5_ and ozone exposure. Geophys. Res. Lett..

[63] Greenstone M., Nilekani J., Pande R., Ryan N., Sudarshan A., Sugathan A. (2015). Lower pollution, longer lives: Life expectancy gains if India reduced particulate matter pollution. Econ. Polit. Wkly..

[64] Health Effects Institute (2018). Burden of Disease Attributable to Major Air Pollution Sources in India. GBD MAPS Working Group, Summary for Policy Makers.

[65] Ramanathan K. (2003). The Energy and Resources Institute National Energy Map for India: Technology Vision 2030 (Summary for Policy-Makers).

[66] Bush K.F., Luber G., Kotha S.R., Dhaliwal R.S., Kapil V., Pascual M., Brown D.G., Frumkin H., Dhiman R.C., Hess J. (2011). Impacts of Climate Change on Public Health in India: Future Research Directions. Environ. Health Perspect..

[67] Anenberg S.C., Belova A., Brandt J., Fann N., Greco S., Guttikunda S., Heroux M., Hurley F., Krzyzanowski M., Medina S. (2016). Survey of ambient air pollution health risk assessment tools. Risk Anal..

[68] Fann N., Kim S.-Y., Olives C., Sheppard L. (2017). Estimated Changes in Life Expectancy and Adult Mortality Resulting from Declining PM_2.5_ Exposures in the Contiguous United States: 1980–2010. Environ. Health Perspect..

[69] Maji K.J., Arora M., Dikshit A.K. (2017). Burden of disease attributed to ambient PM_2.5_ and PM_10_ exposure in 190 cities in China. Environ. Sci. Pollut. Res..

[70] Heo J., Adams P.J., Gao H.O. (2016). Reduced-form modeling of public health impacts of inorganic PM_2.5_ and precursor emissions. Atmos. Environ..

[71] Nasari M.M., Szyszkowicz M., Chen H., Crouse D., Turner M.C., Jerrett M., Pope C.A., Hubbell B., Fann N., Cohen A. (2016). A class of non-linear exposure-response models suitable for health impact assessment applicable to large cohort studies of ambient air pollution. Air Qual. Atmosphere Health.

[72] Laumbach R.J., Kipen H.M. (2012). Respiratory health effects of air pollution: update on biomass smoke and traffic pollution. J. Allergy Clin. Immunol..

[73] Guarnieri M., Balmes J.R. (2014). Outdoor air pollution and asthma. Lancet.

[74] Jain V., Dey S., Chowdhury S. (2017). Ambient PM_2.5_ exposure and premature mortality burden in the holy city Varanasi, India. Environ. Pollut..

[75] Balakrishnan K., Cohen A., Smith K.R. (2014). Addressing the Burden of Disease Attributable to Air Pollution in India: The Need to Integrate Across Household and Ambient Air Pollution Exposures. Environ. Health Perspect..

[76] Samet J.M., Zeger S.L., Dominici F., Curriero F., Coursac I., Dockery D.W., Schwartz J., Zanobetti A. (2000). The National Morbidity, Mortality, and Air Pollution Study. Part II: Morbidity and Mortality from
Air Pollution in the United States.

[77] Revi A. (2008). Climate change risk: An adaptation and mitigation agenda for Indian cities. Environ. Urban..

[78] Nijman J. (2012). India’s Urban Challenge. Eurasian Geogr. Econ..

[79] Office of the Registrar General and Census Commissioner, Ministry of Home Affairs (Government of India) Census of India—Vital Statistics—Sample Registration System. http://www.censusindia.gov.in/vital_statistics/Vital_Rates/Vital_rates.aspx.

[80] Joshi P., Islam S., Pais P., Reddy S., Dorairaj P., Kazmi K., Pandey M.R., Haque S., Mendis S., Rangarajan S. (2007). Risk factors for early myocardial infarction in South Asians compared with individuals in other countries. J. Am. Med. Assoc..

[81] Duong M., Islam S., Rangarajan S., Teo K., O’Byrne P.M., Schünemann H.J., Igumbor E., Chifamba J., Liu L., Li W. (2013). Global differences in lung function by region (PURE): An international, community-based prospective study. Lancet Respir. Med..

[82] Fulambarker A. (2010). Comparison of Pulmonary Function in Immigrant vs US-Born Asian Indians. CHEST J..

[83] Smith K.R., Peel J.L. (2010). Mind the Gap. Environ. Health Perspect..

